# A bioassay system of autologous human endothelial, smooth muscle cells, and leukocytes for use in drug discovery, phenotyping, and tissue engineering

**DOI:** 10.1096/fj.201901379RR

**Published:** 2019-12-05

**Authors:** Blerina Ahmetaj‐Shala, Ryota Kawai, Isra Marei, Zacharoula Nikolakopoulou, Chih‐Chin Shih, Bhatti Konain, Daniel M. Reed, Róisín Mongey, Nicholas S. Kirkby, Jane A. Mitchell

**Affiliations:** ^1^ Cardiothoracic Pharmacology National Heart and Lung Institute Imperial College London London UK; ^2^ Medicinal Safety Research Laboratories Daiichi‐Sankyo Co. Ltd. Tokyo Japan; ^3^ Qatar Foundation Research and Development Division Doha Qatar; ^4^ Centre for Haematology Faculty of Medicine Imperial College London London UK; ^5^ Department of Pharmacology National Defense Medical Center Taipei R.O.C., Taiwan

**Keywords:** blood vessel, cytokine response, stem cells

## Abstract

Blood vessels are comprised of endothelial and smooth muscle cells. Obtaining both types of cells from vessels of living donors is not possible without invasive surgery. To address this, we have devised a strategy whereby human endothelial and smooth muscle cells derived from blood progenitors from the same donor could be cultured with autologous leukocytes to generate a same donor “vessel in a dish” bioassay. Autologous sets of blood outgrowth endothelial cells (BOECs), smooth muscle cells (BO‐SMCs), and leukocytes were obtained from four donors. Cells were treated in monoculture and cumulative coculture conditions. The endothelial specific mediator endothelin‐1 along with interleukin (IL)‐6, IL‐8, tumor necrosis factor α, and interferon gamma‐induced protein 10 were measured under control culture conditions and after stimulation with cytokines. Cocultures remained viable throughout. The profile of individual mediators released from cells was consistent with what we know of endothelial and smooth muscle cells cultured from blood vessels. For the first time, we report a proof of concept study where autologous blood outgrowth “vascular” cells and leukocytes were studied alone and in coculture. This novel bioassay has usefulness in vascular biology research, patient phenotyping, drug testing, and tissue engineering.

**Abbreviations**
α‐SMA: α‐smooth muscle actin
BOECs: blood outgrowth endothelial cells
BO‐SMCs: blood outgrowth smooth muscle cells
ET‐1: endothelin‐1
FBS: fetal bovine serum
FCS: fetal calf serum
IFN‐γ: interferon‐γ
IL‐6: interleukin‐6
IL‐8: interleukin‐8
IP10: interferon gamma‐induced protein 10
PBMCs: peripheral blood mononuclear cells
TNF‐α: tumor necrosis factor‐α

## 1 INTRODUCTION

Blood vessels are comprised of endothelial cells which line the luminal surface and the underlying smooth muscle cells which provide the structural component. In healthy blood vessels, the endothelium is the prime site of mediator release while the smooth muscle is an effector cell which responds to paracrine signals released by endothelial cells. For example, within blood vessels the endothelium releases endothelin‐1 (ET‐1) which has little or no effect on the endothelium itself but stimulates smooth muscle to contract and remodel.^1^ Endothelial cells are also important immune cells which release lipid mediators^2^ and cytokines^3^ that amplify inflammatory responses.

In a disease setting, smooth muscle cells do not normally release mediators in healthy vessels, whereas they can be “induced” to release vasoactive autacoids including ET‐1^4^, ^5^ and inflammatory cytokines.^6^, ^7^ Thus, while endothelial cells are critically important in health and disease, the underlying smooth muscle is also a key player in inflammatory responses and as such is often the major site of pathology and drug action.

Much of what we know about endothelial and smooth muscle cells, particularly in regard to inflammatory responses and mechanism of disease, has been achieved using isolated cells in culture obtained from blood vessels. However, since blood vessels are inaccessible and can only be obtained during surgery or postmortem, there are obvious limitations in what vascular cells derived from arteries or veins can deliver as research and therapeutic tools. These limitations can be overcome by using vascular cells derived from stem cells or from progenitor cells^8^; a popular example of which are the blood outgrowth endothelial cells (BOECs), also known as late outgrowth endothelial cells.^9^, ^10^ BOECs are easily grown out from peripheral blood mononuclear cells (PBMCs) emerging as well‐defined colonies after 7‐21 days in culture.^10^, ^11^ As such, BOECs have been extensively studied and have provided a noninvasive means to study patient endothelial cells for biomarkers, mechanistic studies, and in tissue engineering. Similarly, although less well described, there is a smooth muscle cell type which can also be grown out from blood cells.^12^ In this way, blood outgrowth smooth muscle cells (BO‐SMCs) have the potential to provide the same value as BOECs in vascular cell research.^13^, ^14^, ^15^, ^16^, ^17^

It is well known that vascular cells interact with leukocytes to amplify inflammatory responses. An important example in drug testing is the fact that cytokine storm responses to biological drugs such as the CD28 superantigen, TGN1412, in aqueous phase require leukocytes and an endothelial interface.^18^ However, most of our current knowledge of leukocyte and endothelial cell interactions has been obtained using endothelial cells from one donor (eg, human umbilical vein endothelial cells; HUVEC) and PBMC/leukocytes from another, which introduces obvious confounding factors related to tissue mismatching and where HUVECs are used, immune privilege. To address this for endothelial cells, we have developed a bioassay using BOECs in coculture with autologous leukocytes to generate a “same donor” platform for drug testing.^19^ Although for a fuller understanding of drug action, patient phenotyping, and vascular inflammation, it is essential that the vascular smooth muscle component be also considered.

We suggest that the use of BO‐SMCs along with autologous BOECs and leukocytes can provide a comprehensive vascular bioassay system. This is important since without such an approach it is not possible to identify which cell type(s) (endothelial smooth muscle and/or leukocytes) are dysfunctional in vascular disease. Moreover, using blood outgrowth cells, it is possible to compare responses of autologous cells in cumulative culture from matched healthy control donors with those from individuals with cardiovascular and other diseases. However, these ideas have not previously been tested.

Here for the first time, we report a proof of concept study where autologous blood outgrowth cells and PBMCs were studied in cumulative coculture platings to generate a same donor “vessel in a dish” bioassay platform. Vasoactive and inflammatory mediators were measured under control and inflammatory conditions in culture.

## 2 MATERIALS AND METHODS

Blood was donated from 23 donors collected from a total of 28 donations (some donors donated more than once). All subjects provided written informed consent; local ethical approval was obtained from NRES Committee London ‐ West London & GTAC (15/LO/0223).

### 2.1 PBMC cryopreservation and revival

Blood (40‐50 mL) was collected into 8‐mL vacutainers containing anticoagulant (BD Biosciences, Oxford, UK) and diluted 1:1 with room temperature serum‐free RPMI (without glutamine; Gibco, UK) media and tubes inverted gently to mix. The diluted blood was then layered into falcon tubes containing histopaque (Sigma Aldrich, Gillingham, UK) at room temperature and centrifuged at 800 g for 20 minutes without deceleration (acc.9, dec.0). The supernatant was removed and the PBMC layer (or “buffy coat”) from each tube was carefully transferred to a 50‐mL falcon tube and the volume adjusted to 40 mL using serum‐free RPMI medium. The tubes were then centrifuged (450 *g*) for 10 minutes at 20°C with deceleration (acc.9, dec.3). Cell pellets were combined into one tube and resuspended in 20 mL of serum‐free RPMI and cells counted on a hemocytometer. The cells were then centrifuged (450 *g* for 10 minutes;) before being resuspended in fetal calf serum (FCS; Sigma Aldrich, Gillingham, UK containing 10% DMSO (Sigma Aldrich, Gillingham, UK) in a concentration of approximately 10^7^ cells/mL and kept at −80°C for 18‐72 hours before being transferred to liquid nitrogen.

On the day of the experiment, PBMCs were removed from liquid nitrogen and thawed at 37°C. The cell suspension was transferred to a 15‐mL falcon tube containing 5 mL of serum free RPMI and centrifuged (450 *g*) for 10 minutes at 20°C. The supernatant was removed and 3 mL of RPMI containing 1% donor serum (obtained by collecting blood in vacutainers containing silica and gel [BD Biosciences, Oxford, UK] which was left to stand at 30 minutes at room temperature and then centrifuged at 1300 *g* for 10 minutes) was added and the cells counted. In separate experiments designed to test the effect of cryopreservation on PBMC viability and functional responses, freshly elicited and cryopreserved cells from six separate donors were plated in parallel and either left unstimulated or activated with LPS (1 μg/mL) for 24 hours. Viability was measured using the Alamar Blue assay and release of interleukin (IL‐8) and TNF‐α measured by ELISA as described below. Cryopreserved PBMCs had a moderate but statistically significant lower cell viability when compared to freshly elicited cells. However, release of TNF‐α and IL‐8 were similar from cryopreserved and freshly elicited cells (Figure S1).

### 2.2 Blood outgrowth vascular cells

BOECs and BO‐SMCs were grown as described previously with the following modifications.^11^, ^20^ Human peripheral blood samples (50 mL) were collected from healthy donors in BD Vacutainer Cell Preparation Tubes containing Sodium Heparin/Ficoll (BD Biosciences, UK). PBMCs were separated by centrifugation at 1600 RCF for 30 minutes at room temperature, followed by three washing steps with PBS and 10% FBS, and were centrifuged at 520 RCF for 10 minutes after each wash. PBMCs were then resuspended in EGM‐2 media, and cells were seeded at a density of 3 × 10^7^ cells/well into six‐well plates previously coated with type I rat tail collagen (50 μg/mL) and maintained in an incubator at 37°C in an atmosphere of 95% air: 5% CO_2_. Non‐adherent cells were carefully aspirated after 24 hours of culture, and the wells were washed slowly once with EGM‐2 media containing 10% fetal bovine serum (FBS) (Lonza, Slough, UK). Media was replaced with 4 mL of fresh EGM‐2 complete media. Media was changed and cells washed each day for 3 days, and then every 2‐3 days without washing until colonies emerged. For BOEC isolation, only EGM‐2 media was used whereas for BO‐SMC’s EGM‐2 alone or supplemented with PDGF‐β (50 ng/mL) at day 8 was used. These media/supplements were maintained throughout the cell isolation process. In this study, autologous pairs of BOECs and BO‐SMCs from four donors were used: three male and one female (see Results section). BOECs from all four donors and BO‐SMC from three of the four donors were grown using Lonza EGM‐2 media (Lonza, Slough, UK) + 10% FBS and from the fourth donor using Lonza EGM‐2 media (Lonza, Slough, UK) containing PDGF (50 ng/mL) + 10% FBS. After colonies emerged, BOECs and BO‐SMCs proliferated readily and were expanded to collagen‐coated T25 and T75 flasks and used at passages 4‐6.

### 2.3 Cell plating and treatments

BO‐SMCs were suspended in EGM2 media containing 1% human AB‐serum (Cambridge Bioscience, Cambridge, UK) and plated at a density of 10 000 cells in 100 μL in individual gelatine‐coated wells of a 96‐well plate before being placed in an incubator for 24 hours. After this, media was removed and either replaced with 100 μL of fresh media (EGM2 media containing 1% of human AB‐serum) or 10 000 BOECs in 100 μL of EGM2 media containing 1% of human AB‐serum. At the same time, 10 000 BOECs in 100 μL of EGM2 media containing 1% of human AB‐serum were plated onto parallel gelatin‐coated wells of the same 96‐well plate and plates returned to the incubator for 24 hours. To begin the experiment, media was removed from BO‐SMC/BOEC mono‐ and coculture wells and replaced with either 100 000 PBMCs in 180 μL of RPMI containing 1% autologous serum or 180 μL of cell‐free media (RPMI containing 1% autologous serum). At the same time, 100 000 PBMCs in 180 μL of RPMI containing 1% autologous serum was added to gelatin‐coated wells of the same 96‐well plate. After 30 minutes, 20 μL of either vehicle (RPMI containing 1% autologous serum) or tumor necrosis factor α (TNF‐α) and/or interferon‐γ (IFN‐γ) were added to give a final concentration of 10 and 30 ng/mL, respectively. Plates were then placed in a cell culture incubator for 24 hours, after which plates were centrifuged at 450 *g* for 10 minutes and supernatant removed for analysis.

### 2.4 Imaging

Cell images were obtained using a light microscope at ×10 magnification with a Nikon VR ISO3200 camera attachment (Tokyo, Japan) as detailed in the figure legend.

### 2.5 Measurement of mediators

ET‐1, IL‐6, IL‐8, TNF‐α and IFN‐γ–induced protein 10 (IP10) were measured using specific ELISAs from R&D Systems (Abingdon, UK) according to manufactures instructions.

### 2.6 FACS analysis

FACS was performed to identify both cell‐surface (CD31, CD34, and CD45) and intracellular (S100A4 and α‐SMA) antigens. BOECs and BO‐SMCs were grown in EGM2 media containing 10% FBS in 25‐cm^2^ culture flask. At confluence, cells were trypsinized and resuspended in PBS containing 3% FBS and then incubated with anti‐cell surface marker antibodies. Intracellular antigens were exposed using a fixation and permeabilization buffer (BioLegend, San Diego, CA). Antibodies used for FACS in this study included CD31, S100A4, CD45 (BioLegend), CD34 (BD Bioscience, San Jose, CA), α‐smooth‐muscle actin (α‐SMA) (R&D). These antibodies were PE/Cy7‐, FITC‐, PE‐, PerCP‐Cy5.5, or Alexa Fluor 405‐conjugated mouse anti‐human antibodies. Isotype‐matched IgG antibodies were used as a control. Samples were analyzed with a BD LSRFortessa flow cytometer (BD Biosciences) and the FlowJo software (FlowJo, LLC, Ashland, OR).

### 2.7 Statistics & data analysis

Data are shown as the mean ± SEM for n experiments where n is defined as individual wells. For each experiment, cells of 3‐4 individual donors were plated in duplicate wells. Analysis was performed using GraphPad Prism version 7 using a one‐way ANOVA or *t* test as detailed in the figure legend.

## 3 RESULTS

### 3.1 Culture of BOECs and BO‐SMCs: Obtaining autologous cells

**Figure 1:**
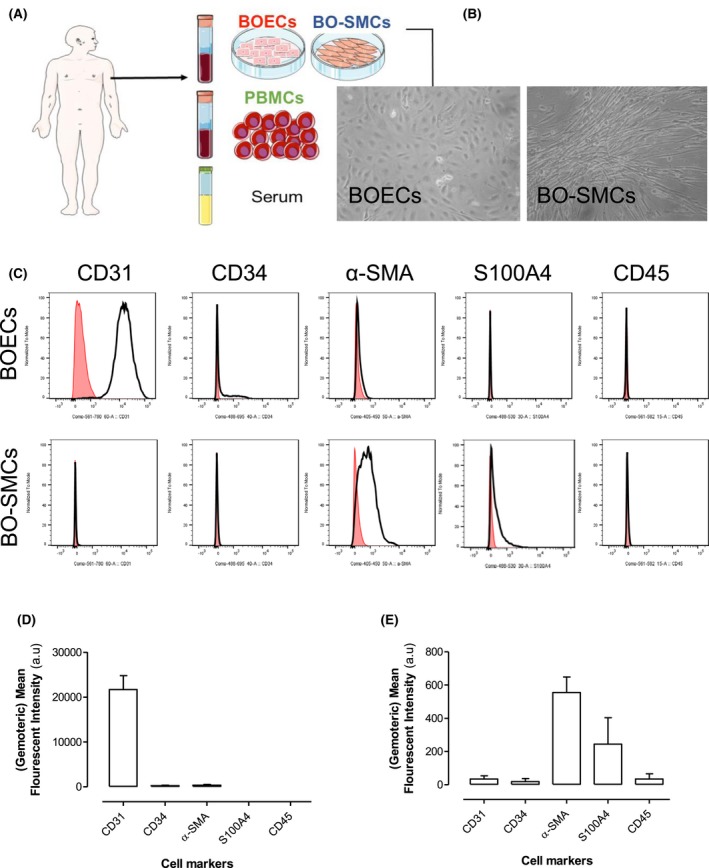
Morphology and cell marker analysis of blood outgrowth endothelial cells (BOECs) and blood outgrowth smooth muscle cells (BO‐SMCs). Schematic of cell collection (A), light microscope images of BOECs and BO‐SMCs (B) were taken at ×10 magnification. Representative cell marker expression panels obtained by FACS analysis of CD31, CD34, α‐SMA, S100A4, and CD45 in BOECs and BO‐SMCs. (C) The open lined histogram represents the test antibodies (anti‐CD31, anti‐CD34, anti‐αSMA, anti‐S100A4, and anti‐CD45). Red filled histograms are the isotype‐matched control IgG antibodies. Pooled results from FACS analysis (from four donors) of BOECs (D) and BO‐SMCs (E) and are shown as the Geometric mean fluorescent intensity minus the associated isotype controls.

BOECs are now routinely grown in numerous laboratories with well‐defined protocols and relatively high success rates. In this study, BOECs were obtained from ≈75% of the 28 donations (from 22 healthy volunteers). BO‐SMCs are a relatively novel cell type and the success rate of growing out these cells is rarely reported. However, a recent paper from Kang and co‐workers commented that BO‐SMCs were successfully obtained from ≈20% of healthy volunteers.^21^ In line with this, in our study, BO‐SMCs were successfully obtained from 22% of donations. Consequently, BOECs *and* BO‐SMCs were successfully obtained from four separate individuals as well as their PBMCs and serum (Figure 1A). The first appearance of outgrowth cells, after initial plating of PBMCs, occurred from day 8 to day 23. On average, BOEC colonies appeared on day 15 and BO‐SMC colonies on day 11. BOECs and BO‐SMCs were initially identified by typical endothelial cell (cobblestone) and smooth muscle cell (fusiform) morphologies, respectively (Figure 1B). BOECs expressed relatively high endothelial (CD31) but low smooth muscle or fibroblast (α‐SMA, S100A4) cell markers, while BO‐SMCs expressed relatively high α‐SMA and low CD31, with moderate and variable expression of S100A4. The expression of the hemopoietic cell marker, CD34, and the leukocyte marker, CD45, was relatively low in both cell types (Figure 1C‐E).

### 3.2 ET‐1 release by BOECs, BO‐SMCs, and PBMCs in mono‐ and coculture conditions

**Figure 2:**
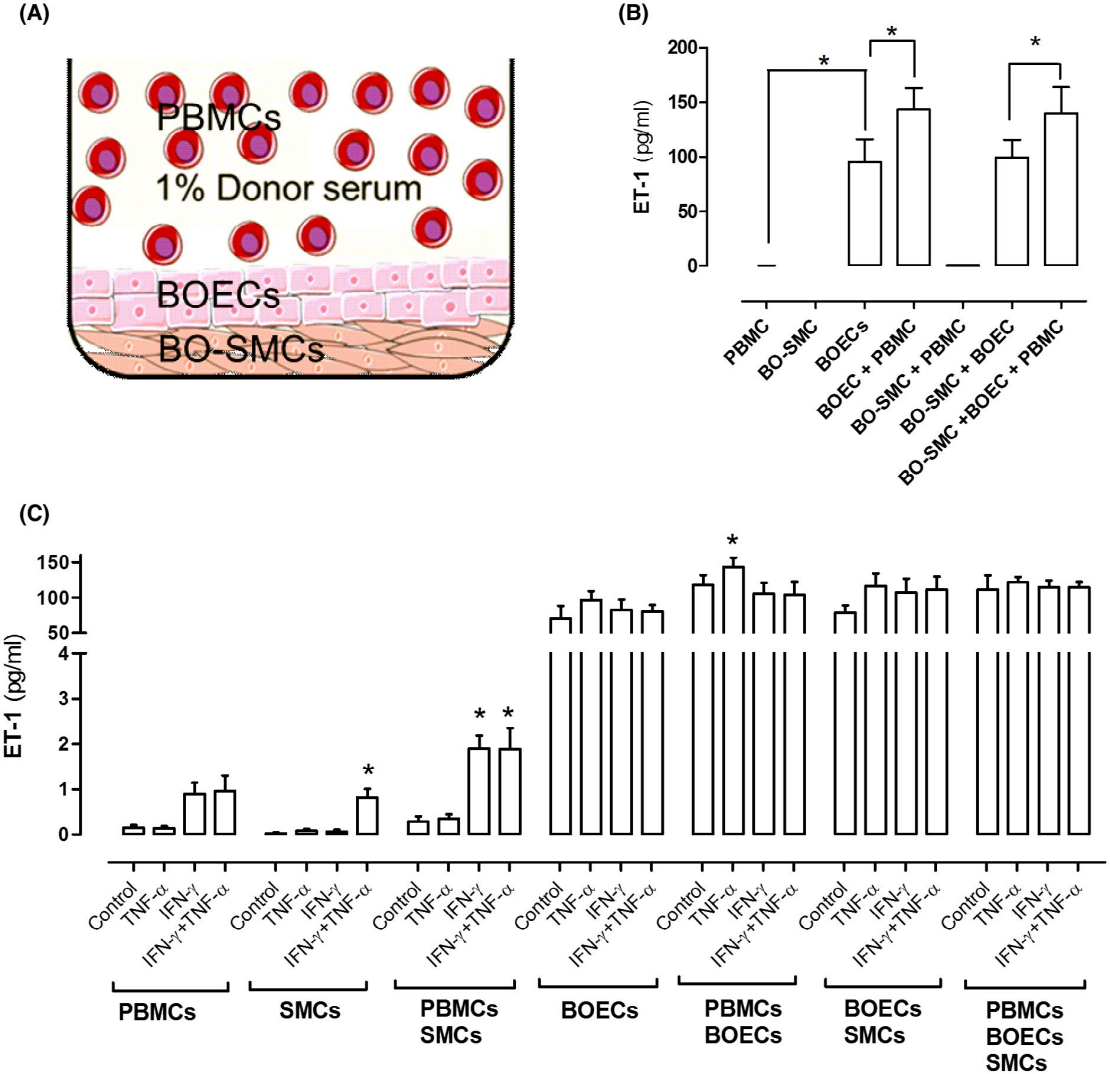
Endothelin‐1 (ET‐1) release from autologous peripheral blood monocytes (PBMCs), blood outgrowth endothelial cells (BOECs), and blood outgrowth smooth muscle cells (BO‐SMCs) in monoculture and in cumulative coculture. Schematic of cells in culture well (A) ET‐1 release from cells under basal conditions (B) and after stimulation with TNF‐α at 10 ng/mL and/or IFN‐γ at 30 ng/mL (C). Data are mean ± SEM for eight incubations of cells from four donors (A) and six incubations of cells from three donors (C). Data were analyzed using one‐way ANOVA followed by multiple comparisons test (B) or one‐way ANOVA followed by Dunnett's post‐test where treatments within each condition were compared to control (C)

In culture, endothelial cells from all origins, including BOECs, release high levels (relative to other cell types) of ET‐1.^22^ Indeed, the release of high levels of ET‐1 is a unique defining feature of endothelial cells. As expected, therefore, the BOECs used in our coculture model (Figure 2A) released high levels of ET‐1 (Figure 2B). By contrast, vascular smooth muscle cells release negligible levels of ET‐1, unless stimulated with the specific combinations of IFN‐γ plus TNF‐α.^23^, ^24^ This feature of vascular smooth muscle cells distinguishes them from lung fibroblasts, which do not release ET‐1 when stimulated with TNF‐α and IFN‐γ.^24^ Consistent with this BO‐SMCs released very low levels of ET‐1 under control culture conditions and were induced to release elevated levels when stimulated with IFN‐γ plus TNF‐α (Figure 2C). ET‐1 released by monocultures of PBMCs tended to be increased in the presence of IFN‐γ but this did not reach statistical significance (Figure 2C).

**Figure 3:**
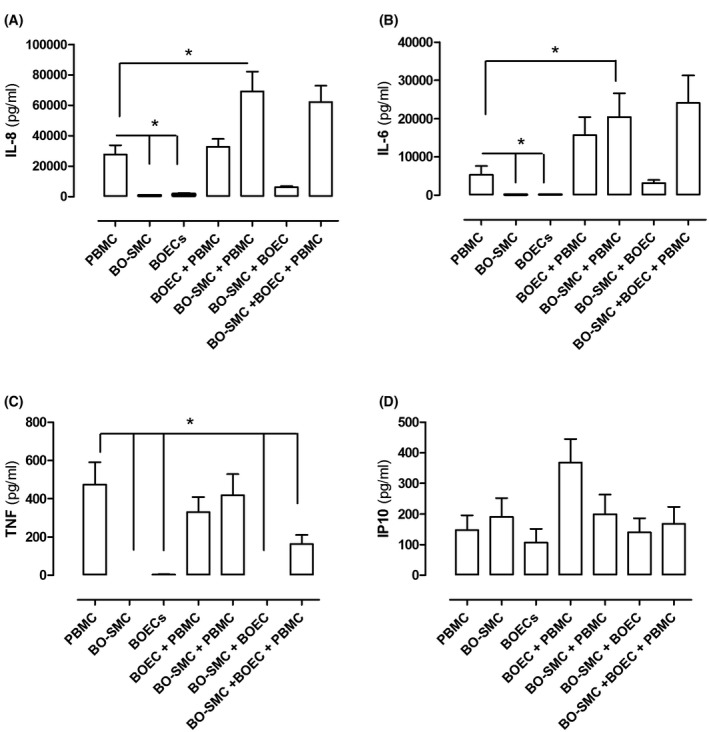
Basal release of IL‐8 (A), IL‐6 (B), TNF‐α (C), and IP10 (D) from autologous peripheral blood monocytes (PBMCs), blood outgrowth endothelial cells (BOECs), and blood outgrowth smooth muscle cells (BO‐SMCs) in monoculture and in cumulative coculture. Data are mean ± SEM for eight incubations from cells of four donors. Data were analyzed using one‐way ANOVA followed by multiple comparisons tests

ET‐1 release by BOECs was not modified by coculture with BO‐SMCs. However, ET‐1 release by BOECs was enhanced in the presence of autologous PBMCs, an effect that persisted in cultures containing all three cell types (Figure 2C). Basal levels of ET‐1 released by BO‐SMCs were not affected by coculture with PBMCs but in the presence of PBMCs, IFN‐γ alone increased ET‐1 release without the requirement for exogenously added TNF‐α. This is likely due to the supply of basally released TNF‐α by PBMCs (Figure 3C).

### 3.3 Cytokine release by BOECs, BO‐SMCs, and PBMCs in mono‐ and coculture conditions

Under basal conditions, monocultures of BOECs and BO‐SMCs released relatively low levels of IL‐8 (Figure 3A) and IL‐6 (Figure 3B) which appeared additive in autologous cocultures of these two cell types. TNF‐α release by monocultures or cocultures of BOECs and BO‐SMCs was negligible (Figure 3C). PBMCs released higher levels of IL‐8, IL‐6, and TNF‐α than BOECs or BO‐SMCs. Release of IL‐8 and IL‐6 by PBMCs was not affected by coculture with BOECs but was increased in cocultures with BO‐SMCs (Figure 3A‐B). TNF‐α release by PBMCs was not affected by coculture with either BOECs or BO‐SMCs but was reduced when PBMCs were combined with both BOECs and BO‐SMCs (Figure 3C). IP10 release was similar from each cell type across all experimental conditions (Figure 3D).

**Figure 4:**
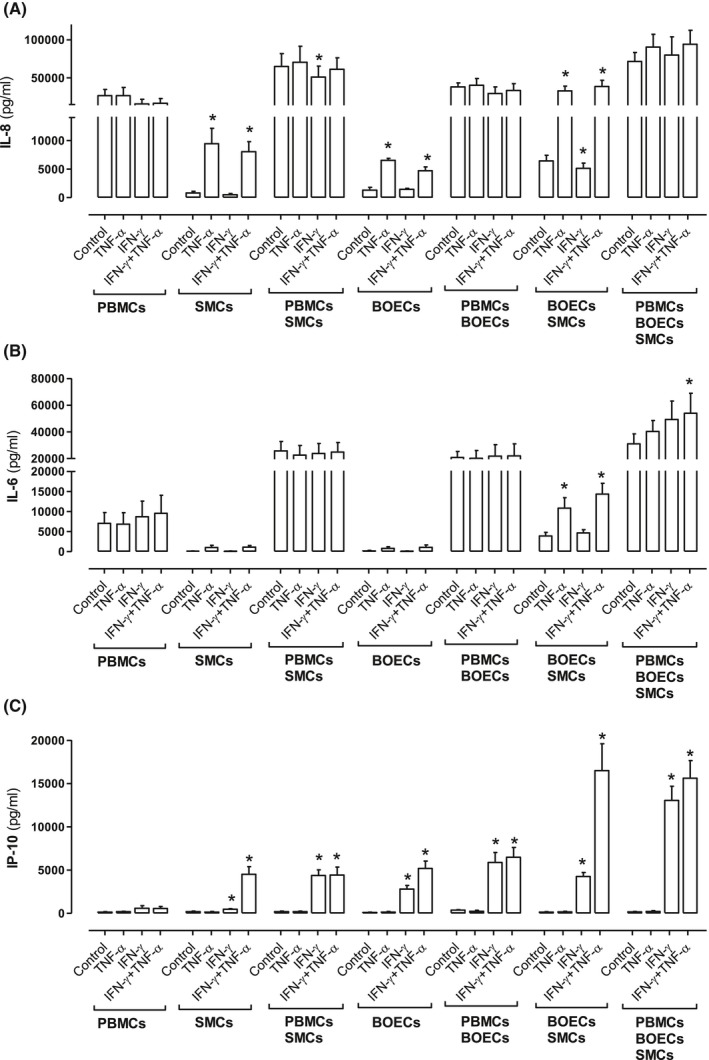
Effect of TNF‐α and/or IFN‐γ on IL‐8 (A), IL‐6 (B), and IP10 (C) release from autologous peripheral blood monocytes (PBMCs), blood outgrowth endothelial cells (BOECs) and blood outgrowth smooth muscle cells (BO‐SMCs) in monoculture and in cumulative coculture. Data are mean ± SEM for six incubations of cells from three donors. Data were analyzed using one‐way ANOVA followed by Dunnett's post‐test where treatments within each condition were compared to control

Monocultures of BOECs and BO‐SMCs, but not PBMCs, released increased IL‐8 when stimulated with TNF‐α but not IFN‐γ. TNF‐α stimulated IL‐8 release by monocultures of BOECs and BO‐SMCs was synergistically enhanced in autologous cocultures of these two cell types. In coculture conditions, IL‐8 release was dominated by the contribution of PBMCs and under these conditions was not further enhanced by either TNF‐α or IFN‐γ (Figure 4A). IL‐6 release was unaffected by either TNF‐α or INF‐γ under monoculture conditions but increased by TNF‐α when BOECs and BO‐SMCs were stimulated in coculture (Figure 4B). Again, as with IL‐8, IL‐6 release was dominated by the contribution of PBMCs when added in coculture to BOECs or BO‐SMCs with no further stimulation seen by IFN‐γ or TNF‐α. However, a modest but significant increase in IL‐6 release was seen in cultures of all three cell types stimulated with the combination of TNF‐α plus IFN‐γ (Figure 4B). IP10 release was not affected by IFN‐γ or TNF‐α in monocultures of PBMCs. However, IP10 release was increased by IFN‐γ in monocultures of BO‐SMCs or BOECs, an effect that was further enhanced by TNF‐α (Figure 4C). Interestingly, IP10 release induced by IFN‐γ alone and IFN‐γ in combination with TNF‐α from monocultures of BOEC and BO‐SMCs was further enhanced when cells were stimulated in coculture (Figure 4C). Similar to ET‐1, IP10 release induced by IFN‐γ from BOECs and/or BO‐SMCs in the presence of PBMCs was not affected by exogenous addition of TNF‐α (Figure 4C).

## 4 DISCUSSION

Here we have exploited “blood outgrowth cell” technology to provide the first demonstration of a bioassay platform where autologous endothelial and smooth muscle cells can be studied alone and in combination with leukocytes from healthy human subjects.

BOECs are well characterized and accepted as bona fide “endothelial” cells with hundreds of publications in the scientific literature. However, human BO‐SMCs are a relatively novel cell type with, to our knowledge, just 11 papers^12^, ^21^, ^25^, ^26^, ^27^, ^28^, ^29^, ^30^, ^31^, ^32^, ^33^ available on PubMed describing them to date. In our hands, BO‐SMCs displayed typical smooth muscle cell morphology with negligible expression of the endothelial marker CD31 and relatively high levels of the smooth muscle marker α‐SMA. BO‐SMCs also expressed the fibroblast marker S100A4. It is not uncommon for SMCs from other origins to co‐express α‐SMA and S100A4^34^ or indeed other fibroblast cell markers. In this regard, it is relatively difficult to truly distinguish vascular smooth muscle cells derived from any source from fibroblasts particularly since fibroblasts express α‐SMA when transitioning to myofibroblasts.^35^ However, BO‐SMCs released increased ET‐1 after stimulation with TNF‐α and IFN‐γ, a feature that we have previously found to distinguish vascular smooth muscle cells from lung fibroblasts.^24^

It was interesting to note that autologous PBMCs could replace the requirement for exogenous TNF‐α and provide cellular conditioning to BO‐SMCs allowing optimal ET‐1 release in the presence of IFN‐γ alone. IFN‐γ induced IP10 release, like ET‐1, is also enhanced by the presence of TNF.^36^ Again, coculture with PBMCs removed the requirement of TNF‐α for IFN‐γ induced IP10 release from either BO‐SMCs or BOECs. These observations illustrate the complex nature of cell‐cell interactions and validate the requirement for coculture bioassay systems that utilize vascular and immune cells in combination. In a pathological setting, these observations are consistent with the idea that leukocytes play a critical role in initiation and propagation of vascular inflammation. In our studies, PBMCs did not directly stimulate cytokine or ET‐1 release from BOECs or BO‐SMCs suggesting that, while they supply TNF‐α, sufficient IFN is not released by PBMCs. This fits with what we know in that IFN is released by dendritic cells which make up only a small fraction of circulating PBMCs. Dendritic cells are, however, located within the vessel wall where they become activated in vascular inflammation associated with infection and atherosclerosis.^37^

In line with what we know about these cell types grown from vessels, BO‐SMCs and BOECs released low levels of cytokines under basal conditions with PBMCs being primary source of IL‐8, IL‐6. and TNF. Points of interest to note from results of unstimulated cocultures included a strong enhanced release of IL‐8 and IL‐6 in cocultures of PBMCs with BO‐SMCs, but not BOECs. These observations support the idea that smooth muscle cells contribute significantly to vascular inflammation.

It is well known that vascular endothelial and smooth muscle cells are activated by cytokines. We found that BO‐SMCs or BOECs released increased IL‐8, IL‐6 and IP10 when stimulated with TNF‐α and/or IFN‐γ, respectively. Interestingly TNF‐α/IFN‐γ stimulated release of these cytokines was dramatically enhanced when BO‐SMC and BOECs were treated in coculture. Again, these observations reiterate the importance of using cocultures to study vascular responses in vitro.

## 5 CONCLUSION

BOECs are a well‐established endothelial cell line. However, BO‐SMCs are less well studied. As such, BO‐SMCs remain somewhat of an enigma in terms of their biology and linage, particularly in respect to how they relate to fibroblasts and myofibroblasts derived from fibroblasts and/or as a result of endothelial‐mesenchymal transition (EMT). Elucidation of the definitive cellular hierarchy of BO‐SMCs is beyond the scope of this study. However, we have successfully isolated BOEC and BO‐SMCs from four individual donors and used them in cumulative cocultures with autologous PBMCs to provide a same‐donor vascular‐bioassay system and validated it in terms of what is currently known about mediator release from endothelial and smooth muscle cells grown from vessels. Once we have a full understanding of the biology and function of BO‐SMCs, this cell type, particularly in combination with autologous BOECs, has the potential to revolutionize vascular biology.

## DISCLOSURES

JAM is inventor on a patent describing the use of autologous vascular cells for assessing cytokine storm responses (WO2014147216A1). RK is an employee of Daiichi‐Sankyo Co.Ltd engaged in development of bioassays to detect cytokine storm reactions. Other authors report no conflicts of interest.

## AUTHOR CONTRIBUTIONS

B. Ahmetaj‐Shala, R. Kawai, I. Marei, C.‐C. Shih, B. Konain, Z. Nikolakopoulou, D.M. Reed, and R. Mongey performed the research; B. Ahmetaj‐Shala, R. Kawai, I. Marei, C.‐C. Shih and B. Konain analyzed the data; B. Ahmetaj‐Shala, R. Kawai, I. Marei, Z. Nikolakopoulou, and N.S. Kirkby edited the paper; J.A. Mitchel, B. Ahmetaj‐Shala, R. Kawai, N.S. Kirkby, and I. Marei designed the research; J.A. Mitchel, B. Ahmetaj‐Shala and I. Marei wrote the paper.
