# Supplementary material for: A bioassay system of autologous human endothelial, smooth muscle cells, and leukocytes for use in drug discovery, phenotyping, and tissue engineering
Source: FASEB J. 2019 Dec 5;34(1):1745–54. doi: 10.1096/fj.201901379RR (PMC6972557; doi:10.1096/fj.201901379RR)
Supplement: Supplementary file 1 [file FSB2-34-1745-s001.pdf]

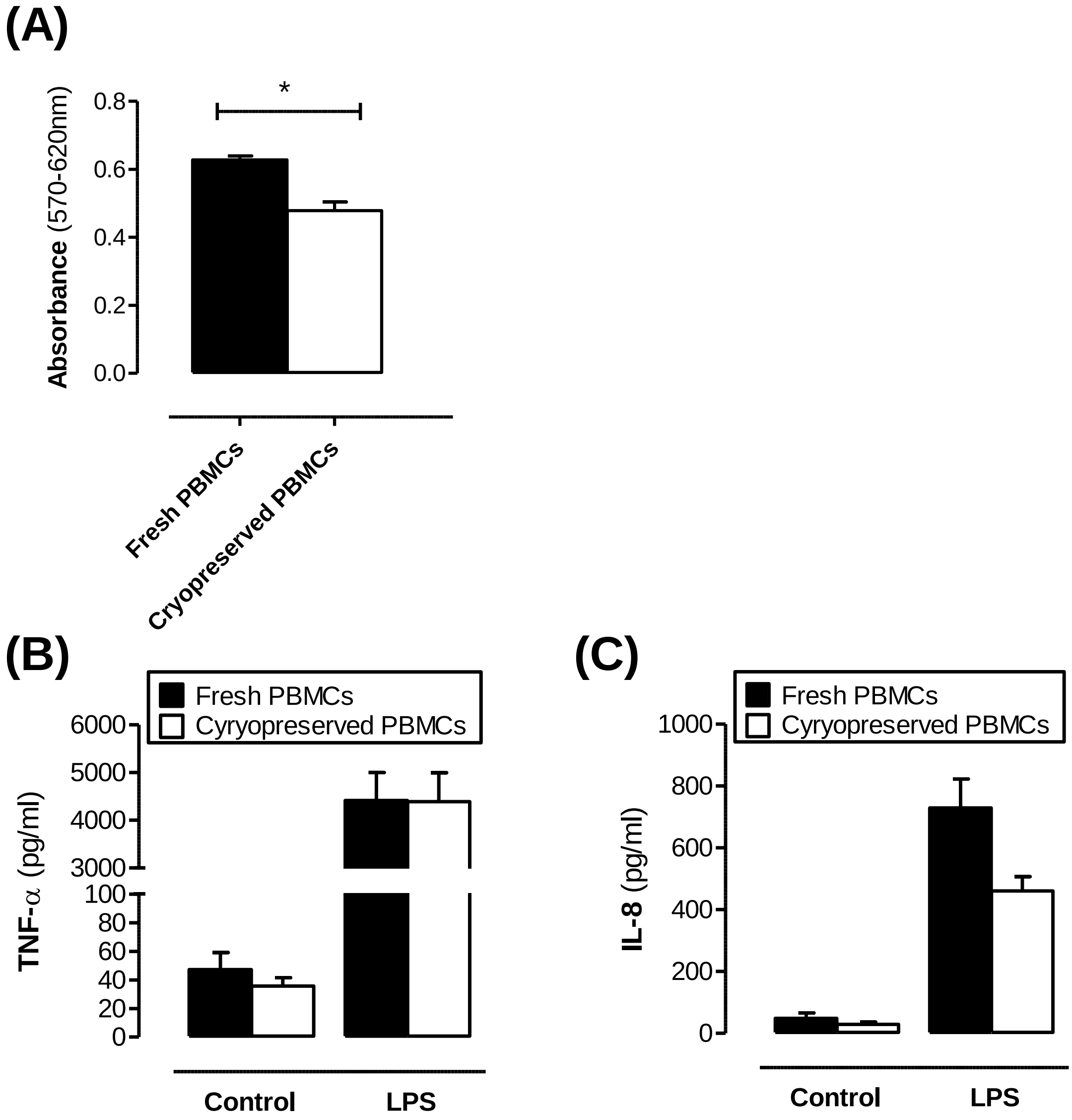

Supplementary Figure 1: Effect of cryopreservation on cell viability (A) and release of TNF-α (B) and IL-8 (C) from human peripheral blood mononuclear cells (PBMCs). Data is the mean ± SEM for cells from n=6 donors. Data was analysed using paired T-test (a) or one-way ANOVA followed by Sidak's multiple comparisons test (b and c); * denotes where $p<0.05$.

Supplementary Figure 1
